# Correction to “Predictors of Recovering Full Consciousness: Results From a Prospective Multisite Italian Study”

**DOI:** 10.1111/ene.70271

**Published:** 2025-07-22

Hakiki, B., Liuzzi, P., Romoli, A.M., Draghi, F., Maccanti, D., De Nisco, A., Burali, R., Toci, T., Grippo, A., Scarpino, M., Mannini, A., Magliacano, A., Estraneo, A., Comanducci, A., Navarro, J., Tramonti, C., Carli, V., Balbi, P., Macchi, C. and Cecchi, F. Predictors of Recovering Full Consciousness: Results From a Prospective Multisite Italian Study. *Eur J Neurol*. 2025;32:e70138. https://doi.org/10.1111/ene.70138.

In the article above, an author's name was incorrectly published as Chiara Tramonti. It should have been Caterina Tramonti.

We apologize for this error.

